# Prevalence of psychiatric and sleep disorders and their impact on quality of life in children with hypermobile Ehlers-Danlos syndrome: an observational study

**DOI:** 10.1007/s00296-025-05836-0

**Published:** 2025-03-25

**Authors:** Amanda K. Hertel, Jordan T. Jones, Ashley Lytch, Emily Cramer, Ariana Schroeder, William R. Black

**Affiliations:** 1 University of Kansas School of Medicine, Kansas City, KS USA; 2 Children’s Mercy Kansas City, Kansas City, MO USA; 3 Nationwide Children’s Hospital, Columbus, OH USA; 4 The Ohio State University College of Medicine, 700 Children’s Dr, Columbus, OH 43205 USA

**Keywords:** Mental health, Sleep disorders, Psychiatric disorders, Pediatrics, Ehlers Danlos syndrome

## Abstract

Hypermobile Ehlers-Danlos Syndrome (hEDS) is the most common connective tissue disorder. However, few studies exist on psychiatric and sleep disorders in pediatric patients with hEDS. This study aims to describe psychiatric and sleep disorders and evaluate their impact on health-related quality of life (HRQoL) in pediatric patients with hEDS. As part of a longitudinal study, a convenience sample of 123 pediatric patients with hEDS, were recruited at a hEDS multidisciplinary clinic in sequential order over a seven-month period. Patient-reported outcomes were completed (Patient Reported Outcomes Measurement Information System Pediatric Profile Version 2 [PROMIS], Generalized Anxiety Disorder-7 [GAD-7], Adolescent Sleep Wake Scale [ASWS], and Pediatric Quality of Life Inventory Rheumatology Module [PEDS-QL Rheum]). The mean age was 15.8 years (*SD* = 2.7), the majority were female (92%) and Caucasian (92%). Most (86%) had at least one psychiatric diagnosis, with anxiety (80%) the most common, followed by depression (42%). Many (42%) also reported poor sleep. Correlations were seen between the GAD-7, PROMIS pain and HRQoL. Worse anxiety and depression were correlated with more sleep problems. Depression, GAD-7, and ASWS were also correlated with worse HRQoL. Performing regression analyses found anxiety and pain to be most predictive of HRQoL. Psychiatric and sleep disorders are prevalent in pediatric patients with hEDS and impact HRQoL negatively. Clinical focus on the anxiety and pain are important due to their impact on HRQoL.

**Supplementary Information:**

The online version contains supplementary material available at 10.1007/s00296-025-05836-0.

## Introduction

The Ehlers-Danlos Syndromes (EDS) are a group of connective tissue disorders with primary features of skin hyperextensibility and joint hypermobility [1]. Hypermobile Ehlers-Danlos Syndrome (hEDS) is the most common of the 13 sub-types of the Ehlers-Danlos Syndromes (EDS) [1] and is associated with higher rates of anxiety and depression than in the general population [2]. Around 70% of individuals with Hypermobility spectrum disorder (HSD) or hEDS have an anxiety disorder and/or depression [3]. Rates of anxiety and depression are similar across other EDS subtypes, which range between 61 − 75% and 22–49% respectively [4, 5]. Higher pain severity is associated with depression in all EDS subtypes [5], but the relationship between pain severity and anxiety is less clear [5]. However, higher anxiety is associated with worse depression, fatigue, and pain in individuals with hEDS [6].

People with HSD and EDS are at increased risk to develop other psychiatric disorders, such as bipolar disorder, attention deficit hyperactivity disorder (ADHD), obsessive-compulsive personality disorder (OCPD), and attempt suicide [7]. The rate of psychiatric disorders is noted to be higher in those with HSD and EDS compared to the general population [8], and there is a high rate of suicidality and previous suicide attempts in women with hEDS [9]. Sleep disturbances are also associated psychiatric disorders in both the general adult [10] and pediatric populations [11]. Sleep difficulties are commonly reported in those with hEDS [12] and are also associated with chronic fatigue [12]. Adult patients with hEDS are five times more likely to have a significant sleep disorder compared to those without hEDS [13], and sleep disorders are associated with poorer functioning and lower quality of life (QoL) [13].

While the rates of anxiety, depression, and sleep disorders are well-studied in adults with hEDS, few studies exist in pediatric patients with hEDS. However, there is some evidence to suggest that higher anxiety and depression are associated with greater functional disability and lower levels of social support [14]. Overall poorer mental health and health-related quality of life (HRQOL) has also been found in pediatric patients with hEDS and other heritable connective tissue disorders [15]. Furthermore, more physical symptoms such as pain, fatigue, and muscle weakness, may also be associated with more anxiety and depression [16]. Similar to adults with hEDS, psychiatric disorders co-occur in pediatric patients, and include ADHD and autism spectrum disorder (ASD) [17]. Sleep concerns have also been observed in many pediatric patients with hEDS [17], and in those with chronic pain [18, 19]. Formal sleep conditions are also common in pediatric patients with hEDS, including insomnia (22%), obstructive sleep apnea (OSA; 26%), periodic limb movement disorder (PLMD) (17%), and hypersomnia (15%) [20], and many (65%) require pharmacologic treatment or referral to behavioral sleep medicine (29%) [20]. While these studies have examined individual relationships between hEDS, pain, psychiatric disorders, and sleep, no study has examined all these factors concurrently.

The objective of this study is to evaluate the prevalence of mental health and sleep disorders and their impact on HRQoL in children and young adults with hEDS. Specifically, this study evaluated relationships and predictive values between anxiety, depression, fatigue, sleep, pain, pain interference, and HRQoL. We hypothesize that higher levels of pain, anxiety, depression, and sleep concern are associated with lower HRQoL. Additionally, this study explores which variables are most impactful on HRQoL when considered concurrently. It was anticipated that in addition to being individually impactful on HRQoL, all variables would remain uniquely important when considered together. In summary, it is hypothesized that greater difficulties with depression, anxiety, and poor sleep will correlate with poorer HRQoL, with anxiety and depression being most predictive based on existing literature.

## Methods and materials

### Study population

As part of a longitudinal study, a convenience sample of 123 pediatric patients with hEDS were recruited at a hEDS multidisciplinary clinic in sequential order between May 2022 and December 2022. The inclusion criteria included a hEDS diagnosis (as defined by the 2017 International Classification for the Ehlers Danlos Syndromes [1]) and an age of less than 22 years at time of diagnosis. This specific multidisciplinary pediatric clinic cares for patients up to age 22. Additionally, as EDS is a rare disease, all eligible patients seen in this clinic were included to be as reflective of our clinic sample as possible. Demographics, psychiatric, and sleep history were collected, and patients completed various patient-reported outcomes (PROs). The PROs included the Patient Reported Outcomes Measurement Information System Pediatric Profile Version 2 (PROMIS), Generalized Anxiety Disorder-7 (GAD-7), Adolescent Sleep Wake Scale (ASWS), and Pediatric Quality of Life Inventory Rheumatology Module (PEDS-QL Rheum).

### Measures

*Clinical Assessments* Clinical psychiatric, sleep symptoms and diagnoses were obtained from the medical record and recorded as binary variables.

*Patient Reported Outcomes Measurement Information System (PROMIS) Pediatric-25 Profile v2.0.* The PROMIS assesses HRQoL through four questions in each domain (anxiety, depression, mobility, fatigue, peer interactions, and pain interference). The questions use a five-point Likert scale, which is then converted into t-scores with higher values representing more of the domain of interest. This measure has been applied to pediatric patients with joint pain [21]. PROMIS sub-scales are calculated so that the population mean score is 50 [22].

*Generalized Anxiety Disorder-7 (GAD-7).* The GAD-7 is a 7-item measure that assesses overall anxiety symptoms and uses a summed score in which a higher value represents more anxiety. This scale has specificity and sensitivity for anxiety in pediatric populations [23].

*Adolescent Sleep Wake Scale (ASWS).* The ASWS evaluates sleep with higher scores indicating better sleep quality. The five subscales include going to bed, falling asleep, maintaining sleep, reinstating sleep, and returning to wakefulness. It is used in adolescents and young adults to assess sleep and identify areas of sleep dysfunction [24].

*Pediatric Quality of Life Inventory Rheumatology Module (PEDS-QL Rheum).* The PEDS-QL Rheum evaluates HRQoL in areas of functioning that are often affected by pediatric rheumatological disease, and includes sub-scales of pain and hurt, daily activities, treatment, worry, and communication. This measure has reliability and validity in pediatric rheumatological populations [25].

### Statistical analysis

Descriptive statistics were calculated for demographic patient reported data. The PRO subscales and totals were evaluated for skew and kurtosis using measures of central tendency and then evaluated for non-normality and kurtosis. For continuous variables, Pearson product-moment correlations were utilized. In cases of correlations between both dichotomous and continuous variables, point-biserial correlations were conducted.

Variables that were significantly correlated with HRQoL as defined by the PEDSQLRheum were entered into a multiple linear regression to predict which variables were significantly and uniquely predictive of HRQoL. Regression analysis was done for five models, one for each PEDS-QL Rheum subscale, with four key predictors including GAD-7, PROMIS Depression, ASWS Sleep Score, and PROMIS pain. Control variables included sex at birth, race, ethnicity, and insurance status. Covariates were all dichotomous variables with Male, Non-White, Non-Hispanic, Non-Private Insurance as reference categories. Private and military insurance were combined into one category. All analyses were performed in SAS version 9.4.

The institutional review board (IRB) approved this study (IRB Study ID: 00001628). Participants were provided an informed waiver of consent at the time of enrollment and work was conducted in accordance with the Declaration of Helsinki.

## Results

### Respondent characteristics and demographics

A total of 123 patients met inclusion criteria (*M*_age_ = 15.8, *SD* = 2.7), with the majority being female (77%) and Caucasian (92%). The most common psychiatric diagnoses were anxiety (80%), depression (43%), and attention-deficit/hyperactivity disorder (ADHD) (17%) with most patients (86%) having at least one psychiatric diagnosis. Approximately half had a previous psychology evaluation (47%), and around half (51%) were on a psychiatric medication. Many (44%) reported sleep problems and poor sleep (42%), and a quarter (25%) had a sleep diagnosis. Some (17%) had a history of a previous sleep evaluation and 16% used a sleep aid medication (Table 1).

**Table 1:** Demographics, psychiatric, and sleep history

Demographics (*N* = 123)		
Gender	**N**	**%**
Female	95	77%
Other (Gender nonconforming, nonbinary, or agender)	18	15%
Male	10	8%
Race		
Caucasian/White	113	92%
More than one race	10	8%
Psychiatric History Variables (*N* = 113)		
**Psychiatric Diagnoses**	**N**	**%**
Anxiety	90	80
History of Psychology Evaluation	53	47
Depression	48	43
ADHD	19	17
Suicidal Ideation	8	7
Non-suicidal self-harm	7	6
Eating Disorder	7	6
Mood disorder	6	5
Autism Spectrum	6	5
PTSD	2	2
Number of Psychiatric Diagnoses		
0	16	14
1	20	18
2	34	30
3	16	14
4	11	10
5	14	12
6	1	1
7	0	0
8	1	1
Sleep History Variables (*N* = 114)		
**Sleep Diagnoses**	**N**	**%**
Any Sleep Problem	50	44
Poor Sleep	48	42
History of Sleep Evaluation	19	17
Insomnia	7	6
Sleep disorder	7	6
Sleep apnea	5	4
Restless leg	3	3
**Number of Sleep Diagnoses**	**N**	**%**
0	64	56
1	28	25
2	10	9
3	5	4
4	6	5
5	1	1

### Bivariate correlations

*Depression and Anxiety.* For this cohort, the GAD-7 mean score was 10.54 (*SD* = 6.26) and the ASWS mean score was 2.28 (*SD* = 0.45). Additionally, the means for the PROMIS Anxiety, Depression, Mobility, Fatigue, Peer Interactions, and Pain Interference subscales were 57.46 (*SD* = 10.42), 56.09 (*SD* = 9.47), 32.75 (*SD* = 5.73), 61.91 (*SD* = 9.10), 45.71 (*SD* = 8.75) and 60.49 (*SD* = 7.64), respectively. For the PEDS-QL Rheum subscales of Pain and Hurt, Daily Activities, Treatment, Worry, and Communication, the means were 26.52 (*SD* = 20.92), 72.68 (*SD* = 19.37), 59.20 (*SD* = 22.16), 53.93 (*SD* = 29.09), and 49.25 (*SD* = 28.49) (Table 2). PROMIS Depression was significantly correlated with the GAD-7 (*r* = 0.71, *p* = < 0.0001) and ASWS (*r* = 0.48, *p* = < 0.0001), and medical chart documentation of depression and sleep involvement (*r* = 0.24, *p* = 0.02) were also significantly related. Significant correlations were found between the GAD-7 and both the ASWS (*r* = 0.50, *p* = < 0.0001) and sleep involvement (*r* = 0.22, *p* = 0.02) (Table 3).

**Table 2:** Mean score and standard deviation of the GAD-7, PEDS-QL Rheum scales, PROMIS scales, and ASWS scales

Subscales (*N* = 123)	Mean Score	SD
**GAD-7 Score**	10.54	6.26
**PEDS-QL Rheum scales**		
Pain and Hurt	26.52	20.92
Daily Activities	72.68	19.37
Treatment	59.20	22.16
Worry	53.93	29.09
Communication	49.25	28.49
**PROMIS scales**		
Anxiety	57.46	10.42
Depression	56.09	9.47
Mobility	32.75	5.73
Fatigue	61.91	9.10
Peer Interactions	45.71	8.75
Pain Interference	60.49	7.64
**ASWS scales**		
Going to Bed	2.57	0.63
Falling Asleep	2.43	0.61
Maintaining Sleep	2.34	0.86
Reinstating Sleep	1.62	0.70
Returning to Wakefulness	2.52	0.57
ASWS Total Score	2.28	0.45

1. Higher GAD-7 scores indicate more anxiety

2. Higher PEDS-QL Rheum sub-scale scores indicate more of the domain of interest

3. Higher PROMIS sub-scale scores indicate more of the domain of interest. A t-score is applied so the general population mean score is 50

4. Higher ASWS scores indicate better sleep quality

**Table 3:** Correlations between diagnoses of depression, anxiety, and sleep dysfunction with GAD-7, PEDS-QL rheum, PROMIS, and ASWS

Subscales	DEP	ANX	SLEEP	GAD	*R*-PAIN	*R*-ACT	*R*-TRE	*R*-W	*R*-COM	*P*-ANX	*P*-DEP	*P*-MOB	*P*-FA	*P*-PEER	*P*-PAIN	A-BED	A-FALL	A-MAIN	A-RE	A-WAKE	A-TOTAL
Depression (DEP)	1.00																				
Anxiety (ANX)	0.22*	1.00																			
Sleep Dysfunction (SLEEP)	0.24*	0.20	1.00																		
GAD-7 Score (GAD)	0.39**	0.07	0.22*	1.00																	
**PEDS-QL Rheum scales**																					
Pain and Hurt (R-PAIN)	-0.26*	0.07	-0.23*	-0.46**	1.00																
Daily Activities (R-ACT)	-0.22*	-0.05	-0.17	-0.31	0.42**	1.00															
Treatment (R-TRE)	-0.23*	-0.07	-0.13	-0.47**	0.49**	0.32*	1.00														
Worry (R-W)	-0.29*	-0.14	-0.21*	-0.67**	0.54**	0.39**	0.63**	1.00													
Communication (R-COM)	0.02	-0.05	-0.11	-0.39**	0.21*	0.06	0.47**	0.47**	1.00												
**PROMIS scales**																					
Anxiety (P-ANX)	0.37*	0.10	0.11	0.80**	-0.37**	-0.44**	-0.40**	-0.62**	-0.32*	1.00											
Depression (P-DEP)	0.49**	0.08	0.13	0.71**	-0.39**	-0.32*	-0.43**	-0.56**	-0.31*	0.69**	1.00										
Mobility (P-MOB)	0.27*	0.05	0.11	0.39**	-0.65**	-0.61**	-0.42**	-0.45**	-0.13	0.47**	0.44**	1.00									
Fatigue (P-FA)	0.42**	-0.07	0.07	0.54**	-0.61**	-0.40**	-0.42**	-0.57**	-0.15	0.52**	0.56**	0.62**	1.00								
Peer Interactions (P-PEER)	-0.15	0.08	-0.08	-0.32*	0.26*	0.24*	0.23*	0.25*	0.27*	-0.31*	-0.38**	-0.32	-0.26	1.00							
Pain Interference (P-PAIN)	0.32*	0.06	0.17	0.48**	-0.76**	-0.40**	-0.47**	-0.48**	-0.26*	0.41**	0.39**	0.64**	0.66**	-0.23*	1.00						
**ASWS scales**																					
Going to Bed (A-BED)	0.18	-0.04	0.08	0.27*	-0.06	-0.01	-0.13	-0.17	-0.04	0.21*	0.33*	0.15	0.21*	-0.19*	0.08	1.00					
Falling Asleep (A-FALL)	0.29*	0.07	0.23*	0.25*	-0.29*	-0.04	-0.17	-0.21*	-0.21*	0.16	0.20*	0.22*	0.22*	-0.23*	0.21*	0.37**	1.00				
Maintaining Sleep (A-MAIN)	0.19	0.07	0.08	0.43**	-0.29*	-0.03	-0.08	-0.21*	-0.19*	0.29*	0.35**	0.16	0.20*	-0.14	0.30*	0.29*	0.37**	1.00			
Reinstating Sleep (A-RE)	0.29*	-0.06	0.22*	0.36**	-0.32*	-0.19*	-0.18	-0.29*	-0.02	0.28*	0.37**	0.27*	0.34*	-0.19*	0.32*	0.21*	0.42**	0.29*	1.00		
Returning to Wakefulness (A-WAKE)	0.06	-0.12	0.10	0.28*	-0.24*	-0.15	-0.16	-0.16	-0.22*	0.20*	0.33*	0.28*	0.19*	-0.11	0.22*	0.40**	0.18	0.29*	0.04	1.00	
ASWS Total Score (A-TOTAL)	0.33*	-0.01	0.22*	0.50**	-0.38**	-0.12	-0.21*	-0.32*	-0.21*	0.36**	0.48**	0.32*	0.35**	-0.26*	0.36**	0.64**	0.71**	0.75**	0.63**	0.51**	1.00

* *p* < 0.05, ** *p* < 0.0001

### Multiple regression analyses

Regression analysis for all five PEDS-QL Rheum models show Pain and Hurt (*R*^2^ = 0.66), Daily Activities (*R*^2^ = 0.39), Treatment (*R*^2^ = 0.54), Worry (*R*^2^ = 0.62), and Communication (*R*^2^ = 0.42). Regression analysis showed that the GAD-7 was associated with the PEDS-QL Rheum Treatment (*B* = -1.58, *p* = 0.02), Worry (*B* = -3.98, *p* = < 0.0001), and Communication subscales (*B* = -4.10, *p* = 0.0002). PROMIS Pain was associated with the PEDS-QL Rheum Pain and Hurt (*B* = -1.65, *p* = < 0.0001) and Daily Activities (*B* = -0.92, *p* = 0.02) subscales. While not statistically significant, the PROMIS Pain and PEDS-QL Rheum Treatment subscale did have good effect modification (*B* = -0.84, *p* = 0.06). Otherwise, no other statistically significant associations were found between the PEDS-QL Rheum sub-sections and PROMIS depression or ASWS (Table 4).

**Table 4:** Regression analysis of PEDS-QL Rheum sub-scales, psychosocial, sleep, and demographic variables

PEDS-QL Rheum Scale	Variable	B	95% CI	SE	t Value	*p*	Eta^2^
Pain and Hurt (R^2^ = 0.66)	Intercept	142.22	[92.32, 192.13]	24.71	5.76	**< 0.0001**	
	GAD-7	-0.59	[-1.51, 0.32]	0.45	-1.31	0.20	0.04
	PROMIS Depression	-0.25	[-0.83, 0.34]	0.29	-0.85	0.40	0.02
	ASWS Sleep Score	0.51	[-2.70, 3.72]	1.59	0.32	0.75	0.00
	PROMIS Pain	-1.65	[-2.23, -1.07]	0.29	-5.74	**< 0.0001**	0.45
	Sex at Birth	3.24	[-11.27, 17.74]	7.18	0.45	0.65	0.00
	Race	2.22	[-15.72, 20.16]	8.88	0.25	0.80	0.00
	Ethnicity	-1.19	[-16.77, 14.39]	7.71	-0.15	0.88	0.00
	Insurance	-5.03	[-13.20, 3.15]	4.05	-1.24	0.22	0.04
Daily Activities (R^2^ = 0.39)	Intercept	187.50	[119.92, 255.07]	33.46	5.60	**< 0.0001**	
	GAD-7	-0.63	[-1.87, 0.61]	0.61	-1.03	0.31	0.13
	PROMIS Depression	-0.11	[-0.90, 0.68]	0.39	-0.27	0.79	0.04
	ASWS Sleep Score	2.06	[-2.29, 6.40]	2.15	0.96	0.35	0.01
	PROMIS Pain	-0.92	[-1.71, -0.14]	0.39	-2.37	**0.02**	0.22
	Sex at Birth	-2.39	[-22.02, 17.24]	9.72	-0.25	0.81	0.00
	Race	-3.09	[-27.38, 21.21]	12.03	-0.26	0.80	0.02
	Ethnicity	-14.58	[-35.67, 6.51]	10.44	-1.40	0.17	0.05
	Insurance	-9.13	[-20.20. 1.94]	5.48	-1.67	0.10	0.06
Treatment (R^2^ = 0.54)	Intercept	229.60	[155.16, 304.04]	36.86	6.23	**< 0.0001**	
	GAD-7	-1.58	[-2.94, -0.21]	0.68	-2.34	**0.02**	0.39
	PROMIS Depression	-0.49	[-1.36, 0.38]	0.43	-1.15	0.26	0.05
	ASWS Sleep Score	-0.45	[-5.24, 4.34]	2.37	-0.19	0.85	0.00
	PROMIS Pain	-0.84	[-1.70, 0.03]	0.43	-1.96	0.06	0.17
	Sex at Birth	-6.70	[-28.33, 14.93]	10.71	-0.63	0.54	0.00
	Race	0.84	[-25.92, 27.61]	13.25	0.06	0.95	0.06
	Ethnicity	-31.93	[-55.16, -8.69]	11.50	-2.78	**0.01**	0.16
	Insurance	-0.42	[-12.61, 11.77]	6.04	-0.07	0.94	0.00
Worry (R^2^ = 0.62)	Intercept	173.19	[87.24, 259.15]	42.56	4.07	**0.0002**	
	GAD-7	-3.98	[-5.55, -2.40]	0.78	-5.10	**< 0.0001**	0.59
	PROMIS Depression	0.23	[-0.77, 1.23]	0.50	0.46	0.64	0.00
	ASWS Sleep Score	-4.14	[-9.67, 1.39]	2.74	-1.51	0.14	0.03
	PROMIS Pain	-0.03	[-1.03, 0.97]	0.49	-0.06	0.96	0.02
	Sex at Birth	-20.01	[-44.99, 4.97]	12.37	-1.62	0.11	0.05
	Race	-0.95	[-31.86, 29.96]	15.30	-0.06	0.95	0.02
	Ethnicity	-19.12	[-45.95, 7.70]	13.28	-1.44	0.16	0.05
	Insurance	-3.58	[-17.66, 10.50]	6.97	-0.51	0.61	0.01
Communication (R^2^ = 0.42)	Intercept	192.30	[79.93, 304.67]	55.64	3.46	**0.0013**	
	GAD-7	-4.10	[-6.16, -2.04]	1.02	-4.03	**0.0002**	0.31
	PROMIS Depression	0.67	[-0.64, 1.99]	0.65	1.04	0.31	0.00
	ASWS Sleep Score	-2.28	[-9.50, 4.95]	3.58	-0.64	0.53	0.00
	PROMIS Pain	0.41	[-0.89, 1.72]	0.65	0.64	0.53	0.00
	Sex at Birth	-14.07	[-46.72, 18.58]	16.17	-0.87	0.39	0.00
	Race	-38.08	[-78.49, 2.32]	20.01	-1.90	0.06	0.17
	Ethnicity	-22.91	[-57.98, 12.16]	17.37	-1.32	0.19	0.05
	Insurance	-9.29	[-27.70, 9.11]	9.11	-1.02	0.31	0.02

## Discussion

The overall objective of this study was to quantify rates of psychiatric and sleep dysfunction in pediatric and young adult patients with hEDS and evaluate the impact of mental health and sleep on HRQoL. Previous studies have shown that pediatric patients with hEDS are at increased risk for mental health and psychiatric conditions [17]. This study evaluated several areas of mental health functioning and found that pediatric patients with hEDS have significant mental health issues with much more anxiety than depression. This study also found that many patients have a sleep problem and poor sleep. In this study, both psychiatric disorders and sleep issues are prevalent in pediatric and young adult patients with hEDS. Additionally, depression and anxiety symptoms appear to adversely affect HRQoL, particularly in the domains of pain, hurt, and worry. It was also identified that anxiety and pain are the most predictive of HRQoL in pediatric and young adult patients with hEDS when also considering other psychosocial and health measure.

Many patients in this cohort had a diagnosed psychiatric disorder, which aligns with adult studies and the general population. Our study found that anxiety was the most common diagnosis (80%), which is similar, but higher compared to adults with hEDS and HSD (51–75%) [3, 4, 6]. This higher rate could be explained by increased prevalence in the pediatric and young adult population more broadly [26], and that a subset of these patients were recruited during the COVID pandemic, a period of higher stress and uncertainty [27]. Prior to the COVID pandemic, anxiety and depression prevalence in the general pediatric population were 13% and 12%, respectively [28]. However, anxiety rates during COVID in the general pediatric population were around 21% [29]; thus, the higher rates of anxiety in this sample, nearly 4 times higher than the general population, are unlikely to be attributed to only COVID. Our study also found depression to be prevalent (43%), yet lower than the 70% found in adult hEDS and HSD [3]. This prevalence of depression is still higher than in the general pediatric population during the COVID-19 Pandemic (25.2%) [29]. Nonetheless, the COVID pandemic likely did contribute to increased rates of depression and anxiety seen in this study due to decreased emotional and social connections, virtual classes, disruptions in sleep schedules, increased screen time, and economic concerns among many others [30–32]. As both anxiety and depression are elevated in hEDS, clinicians should be mindful of these areas of concern when seeing patients who have hEDS. Consideration of routine mental health screening in patients with hEDS should be considered.

This study also found rates of ADHD (17%) and autism spectrum disorder (5%) that were similar to other pediatric hEDS studies [17], and still elevated compared to the general population (5% ADHD and 1% ASD) [33]. However, different from adult literature [7, 8],, more serious forms of mental illness (e.g., schizophrenia, bi-polar disorder, and personality disorders) were not observed in this sample. Given that these diagnoses are rarely diagnosed in the pediatric population, and that our sample size consisted primarily of pediatric patients under the age of 18, it is unsurprising that no patients in this study had those diagnoses. A surprising and concerning result from this study was that while most patients (86%) in this study had at least one psychiatric diagnosis, only about half (47%) had a previous psychology evaluation, or were prescribed medication for a psychiatric condition (51%). This suggests a discrepancy in needed versus obtained or available psychological or psychiatric care, and that routine screening and evaluation of mental health should be performed for all patients with hEDS. Mental health clinicians should be integrated into clinics that evaluate and manage patients with hEDS, similar to other pediatric rheumatological conditions [34].

Sleep concerns were also common in this study, with 44% of patients noting a sleep problem, similar to previous reports in pediatric patients with hEDS (52%) [17]. Surprisingly, the rate of sleep apneas in this study (4%) was somewhat lower than expected. Studies on adults with EDS found a much higher obstructive sleep apnea (OSA) frequency at 32% [13]. This discrepancy may be due to the young age of this study population compared to an adult cohort, and that OSA is much less frequent in the pediatric population (1–4%) [35]. However, the rates of OSA in this study (4%) are still lower than other pediatric hEDS studies (7–26%) [17, 20]. One potential explanation is inadequate screening for this condition in our patient population. Given the high levels of fatigue in hEDS, there may be value in routine screening for OSA, particularly for those who are also reported to snore. OSA is associated with numerous adverse outcomes, including excessive daytime sleepiness and decreased quality of life [13] but is responsive to earlier intervention with CPAP and other therapies. Screening, consultation, and treatment referrals for OSA and sleep disorders may also positively impact HRQoL and should be part of standard evaluation and management planning for hEDS [20]. Similar to the discrepancy between mental health prevalence and ongoing treatment, while 44% of patients in our study reported issues with sleep, only 17% had a previous sleep evaluation, and 16% used sleep aid medications. Thus, across the entirety of this study, while many patients have mental health and sleep concerns, most have not been evaluated by a mental health or sleep medicine specialist. There should be a low threshold to refer to appropriate subspecialty care if concerns arise to meet the needs of patients with hEDS.

Our results also demonstrated that higher anxiety and depression were correlated with more sleep problems, consistent with numerous studies in pediatrics [11, 36, 37]. Studies that have also demonstrated fatigue, depressive symptoms, and anxiety are highly correlated in patients with hEDS [6]. It is also important to consider how anxiety, depression, and sleep are correlated to HRQoL. In this study, symptoms of depression were found to have a negative impact on HRQoL. Because of this, it is important to evaluate depressive symptoms in pediatric patients with hEDS and treat accordingly. Interestingly, in this study, the GAD-7, but not the diagnosis of anxiety, also had a negative impact on HRQoL. It is possible that a diagnosis of anxiety captures overall function, but the GAD-7 and PROMIS capture more acute mental health functioning and representative of how the participant was functioning at the time the measures were completed. Additionally, a diagnosis of anxiety may lead to treatment and improvement in anxiety symptoms meaning that those who were previously diagnosed with anxiety had lower GAD-7 at the time it was administered. Finally, while the presence of sleep dysfunction had little correlation to HRQoL in this study, the ASWS was associated with the HRQoL measures (PROMIS and PEDS-QL Rheum). One potential explanation is that the ASWS does a better job of stratifying various areas of sleep dysfunction, while the presence of sleep dysfunction was recorded as a binary variable (yes/no). So, while someone may report sleep dysfunction, the actual degree of symptom burden (as measured by the ASWS), has a better correlation with HRQoL.

Of all these relationships, this study also found that when considered simultaneously in regression analyses, anxiety (GAD-7) and pain (PROMIS Pain subscale) are the most predictive areas of mental health on HRQoL. While many symptom areas are important in the management of hEDS, mental health screening may be among the top priority to address in management of hEDS. Surprisingly, the PROMIS Depression and ASWS had no significant associations to PEDS-QL Rheum subscales in regression analysis. This suggests that depression and sleep, which are correlated to HRQoL, may be less important to understanding overall HRQoL comparatively to anxiety and pain. Of relevance to the practice of pediatricians and rheumatologists, this study suggests mental health screening tools, notably the GAD-7 and PROMIS Pain subscale, should be incorporated into routine hEDS screening and care. The uses of these PROs may help clinicians monitor areas of functioning most predictive of over-all functioning, and in concert with other PRO measures, clinicians may regard patients as scoring high in both anxiety and pain as being at the greatest risk for overall impairment – clinicians may use this information to justify more intensive psychological interventions and supports. It is also noteworthy that for the PEDS-QL Rheum treatment subscale, ethnicity was also found to be predictive, which may speak to underlying health inequities experienced and reported by some patients that should be evaluated in future work.

### Strengths, limitations, and future studies

This was the first study to explore the relationship between mental health and sleep, and their correlation and predictive value to quality of life in pediatric patients with hypermobile Ehlers-Danlos syndrome. Given increasing incidence of joint-hypermobility and hypermobile EDS globally, these findings may have broad impact on how we monitor and assess hEDS. This study consisted of many strengths, including a larger sample size for the content area, and a large battery of well-validated measures of mental health and psychiatric symptoms. Additionally, this research team also had access to EMR data and were able to utilize this information to better understand the mental health and psychiatric symptoms and presentations in hEDS. However, there are also several limitations which might limit generalizability and should be addressed in future work. One such limitation is that the sample in this study may not be representative of a hEDS more broadly. As this study largely included participants seeking care in a multidisciplinary clinic for hEDS, this sample may be biased toward greater symptoms and impairment compared to those not seeking specialized care. Further, this study focused only on individually reported factors. Given that hEDS may impact the family system, such as higher rates of ADHD, depression, and attempted suicide in siblings of those with EDS [7], this study may miss important family-focused, and ecological relationships that may be helpful to understand the factors that affect mental health in pediatric patients with hEDS. It is important to note that a hEDS diagnosis may have impact on other familial, social, and community systems to which patients are connected [38, 39]. Given the suspected heritability of EDS and hEDS, evaluating disease related functioning in other family members may provide a richer understanding of HRQoL. Also, while psychiatric and sleep diagnoses are recorded in the EMR, it cannot be determined whether these are acute or chronic diagnoses, or what stage of treatment has been undertaken to date to address the diagnoses. Future work should focus on using structured interview and other diagnostic tools to more thoroughly assess mental health symptoms. Additionally, these data were collected at one point in time, and patients’ mood as measured by the GAD-7, PROMIS, or PEDSQL-Rheum may vary over time. Further work should be done to evaluate QoL measures and their relationship to mental health and sleep over multiple time points. Additionally, these patients were seen at a single multidisciplinary EDS clinic, and further work at other locations/clinics/medical centers may overall help the generalizability of this study.

## Conclusion

Psychiatric, sleep, and pain concerns are well documented in adults with hEDS, and emerging evidence suggest that these are areas of concern in pediatric patients with hEDS as well. Young patients with a diagnosis of hEDS should be screened for mental health conditions and sleep concerns, as they have a negative impact on HRQoL. Recognition and treatment of these conditions early may lead to improved HRQoL and functioning over time. Use of the GAD-7 and PROMIS Pain to assess PROs should be a strong consideration as they have predictive value on HRQoL.

## Electronic supplementary material

Below is the link to the electronic supplementary material.

Supplementary Material 1

Supplementary Material 2

## Data Availability

Data available within the article or its supplementary materials. Open Data Sharing: Summary data may be available per request via email to the corresponding author (WRB).
